# Assessing predictors of intention to prescribe sick leave among primary care physicians using the theory of planned behaviour

**DOI:** 10.1186/s12875-017-0690-5

**Published:** 2018-01-16

**Authors:** Yogarabindranath Swarna Nantha, Lei Hum Wee, Caryn Mei-Hsien Chan

**Affiliations:** 1 Non-Communicable Disease Clinic, Seremban Primary Care Clinic, Jalan Rasah, 70300 Seremban, Negeri Sembilan Malaysia; 2 Faculty of Health Sciences, Universiti Kebangsaan Malaysia, 50586 Kuala Lumpur, Malaysia

**Keywords:** Theory of planned behaviour, Sick leave prescribing, Intention, Primary care physicians

## Abstract

**Background:**

Providing sickness certification is a decision that primary care physicians make on a daily basis. The majority of sickness certification studies in the literature involve a general assessment of physician or patient behaviour without the use of a robust psychological framework to guide research accuracy. To address this deficiency, this study utilized the Theory of Planned Behaviour (TPB) to specifically gauge the intention and other salient predictors related to sickness certification prescribing behaviour amongst primary care physicians.

**Methods:**

A cross-sectional study was conducted among *N* = 271 primary care physicians from 86 primary care practices throughout two states in Malaysia. Questionnaires used were specifically developed based on the TPB, consisting of both direct and indirect measures related to the provision of sickness leave. Questionnaire validity was established through factor analysis and the determination of internal consistency between theoretically related constructs. The temporal stability of the indirect measures was determined via the test-retest correlation analysis. Structural equation modelling was conducted to determine the strength of predictors related to intentions.

**Results:**

The mean scores for intention to provide patients with sickness was low. The Cronbach α value for the direct measures was good: overall physician intent to provide sick leave (0.77), physician attitude towards prescribing sick leave for patients (0.77) and physician attitude in trusting the intention of patients seeking sick leave (0.83). The temporal stability of the indirect measures of the questionnaire was satisfactory with significant correlation between constructs separated by an interval of two weeks (*p* < 0.05). Attitudes and subjective norms were identified as important predictors in physician intention to provide sick leave to patients.

**Conclusion:**

An integrated behavioural model utilizing the TPB could help fully explain the complex act of providing sickness leave to patients. Findings from this study could assist relevant agencies to facilitate the creation of policies that may help regulate the provision of sickness leave and alleviate the work burden of sickness leave tasks faced by physicians in Malaysia.

**Electronic supplementary material:**

The online version of this article (10.1186/s12875-017-0690-5) contains supplementary material, which is available to authorized users.

## Background

The act of providing sickness leave to patients is a decision faced by most medical practitioners during routine consultations, especially in primary care settings [1–4]. Studies in various countries indicate that general practitioners are reluctant in their role as gatekeepers of sickness leave tasks [5–13]. Considerable dissonance has been documented between patients who consider themselves worthy of sickness leave and the examining physician who fails to detect an objective clinical finding that is consistent with the history that is given [14]. Consequently, studies have shown that the task and process of providing sickness leave can be a source of emotional strain for physicians as they experience conflict with patients and other stakeholders [6–13, 15].

Worldwide, few studies have examined how physicians routinely prescribe sickness leave [16, 17], particularly when this is compared to the widely available literature focusing on the motivations and behaviour patterns of patients obtaining sick leave. Large variations have been found in the way that physicians regularly prescribe sickness leave [18]. Most studies related to sickness certification appear to focus on the descriptive or epidemiological aspects of physician or patient behaviour and lack theoretical grounding [15, 19]. Fewer studies still have attempted to frame an understanding of the attitudes that underlie actual prescribing behaviours on the part of physicians using theories related to health behaviour [20].

**Fig. 1:**
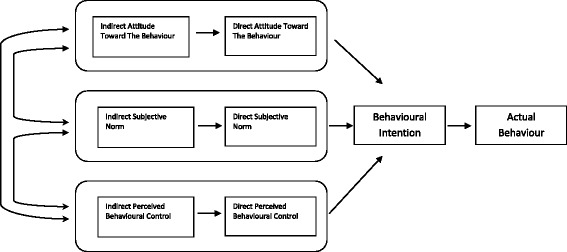
The constructs in the Theory Of Planned Behaviour as proposed by Ajzen (2006)

An in-depth understanding and skillful application of a theoretical framework is essential to any research intervention designed to change patient or physician behaviour [15, 21]. The Theory of Planned Behaviour (TPB) is ideally suited to help acquire valuable information on potential areas for behavioural interventions through this study. The strength of the TPB is that it provides a framework to decipher individuals’ action and allows the researcher to understand the reasons that motivate the behavior under investigation [21].The TPB by Ajzen [22] posits three constructs that determine behaviour intentions: attitude towards behaviour, subjective norm and perceived behavioural control (Fig. 1) [21].

The current study focuses on how these three constructs influence intention to provide sickness certification and actual sick leave prescribing behaviour in clinical practice. This can be measured using both direct and indirect measures. Direct measures are used to obtain a general assessment or opinion of the respondents with either a semantic differential scale or a bipolar disagree-agree scale. Indirect measures serve to examine the respondent’s underlying specific beliefs and outcome evaluations. We hypothesize that attitude, subjective norm or perceived behavioural control are significant predictors of primary care physicians’ intent to provide sickness certification. The objective of this study is to identify the factors that influence physician behaviour in providing sickness leave to patients in a primary care setting.

## Methods

### Study design

A cross-sectional study design was implemented using data from 86 primary health care clinics in two states (Negeri Sembilan and Selangor) in Malaysia consisting of seven and nine districts respectively between February 2014 and March 2015.

### Study population

The suggested sample size for studies using the TPB framework should be more than 160 participants [23]. General practitioners from the clinic can be divided into primary care physicians and family medicine specialists. A total of 329 primary care physicians (out of the 666 physicians who were invited to participate in the study) were recruited. Primary care physicians from all participating primary health care clinics with at least one year of compulsory internship were eligible to participate in the study. Family medicine specialists (consultants) and junior house officers were excluded for two reasons: 1) the frequency of providing sickness certification to patients are vastly minimal compared to all other physicians at the clinic and 2) they attend to a very select number of patients per day and represent a small subset of the primary care physician population in most primary care clinics.

### Survey procedure

A meeting involving representatives from all participating primary health clinics was held to explain the nature of the study. Sets of three envelopes containing instructions and questionnaires for each time point were handed to physicians in charge/clinical administrators during the meeting in a sealed envelope. All questionnaires were self-administered. Written informed consent was obtained from all participating physicians prior to the first data collection.

There were two sets of questionnaires (Q1 and Q2). Q2 was administered two weeks after Q1 was first administered. Additional document file shows these questionnaires in detail (see Additional file 1 and Additional file 2). After a lapse of another two weeks, Q2 was re-administered to gauge temporal stability [23]. Each questionnaire had coded numbers for each participant to ensure de-identification and confidentiality. All answered questionnaires were collected in person by the principal investigator. This project was approved by the Medical Research and Ethics Committee of Malaysia (NMRR 12–1422-14,535).

### Questionnaire design and development

#### Testing of direct measures and elicitation phase

**Fig. 2:**
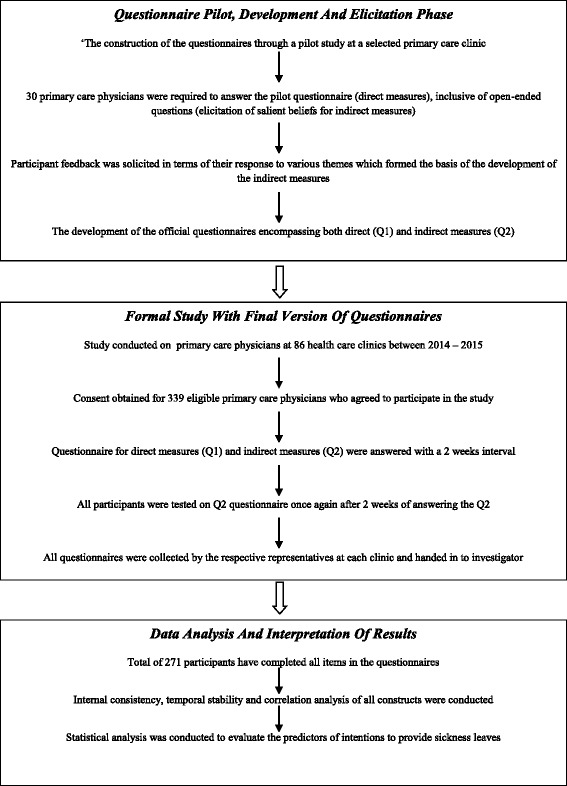
Flow chart of the study design and process of data collection

Figure 2 summarizes the steps taken in conducting the pilot and main study. The development and reliability analysis of the questionnaire was based on guidelines designed for assessments at a primary care setting [23]. In accordance with the general and specific TPB questionnaire guidelines for health services [22, 23] a pilot study was conducted at a single primary care clinic with a sample size of 30 participating physicians.

Items related to the direct measures of TPB were tested during the pilot stage of questionnaire development. Open-ended questions were used to obtain salient beliefs in relation to the TPB constructs. These elicitation questions assessed instrumental attitude, normative attitude, normative influence and perceived behavioural control [21]. This formed the basis to generate the questions related to indirect measures.

Participant feedback was solicited. Each participant was encouraged to state whether, 1) items in the questionnaire were ambiguous or difficult to answer, 2) the questionnaire felt too repetitive or too long, or 3) the questionnaire had less than ideal wording or format. Open responses were likewise solicited from participants. Participants’ response were then categorized according to relevant themes. The extraction of specific themes related to the study was jointly established between authors (YSN and WLH). The two authors obtained consensus on themes derived from the open-ended questions. YSN confirmed the categorization of the identified themes through feedback from individual participants. The preliminary results (recurring themes) were then used to design the indirect measures relevant to the TPB constructs.

#### Description of constructs and questions involving direct and indirect measures

Standard guidelines for TPB were utilized to generate the questionnaire for direct measures [22, 23]. The intention to act or not to act depends on 3 main constructs. These three constructs of TPB are attitude, subjective norm and perceived behavioural control [22].

## Intentions on providing sickness leave

Three items were used to measure intention to provide sickness leave to patients: ‘I plan to provide patients sickness leave during an outpatient consultation; ‘I want to provide patients with sickness leave during an outpatient consultation, ‘I intend to provide patients with sickness leave during an outpatient consultation’. The mean of the response of these three items served as the measure of intention. The scores for this measurement ranged from 1 to 7 (1 = strongly disagree, 7 = strongly agree).

## Attitudes toward providing sickness leave

Two items were utilized in this category: ‘Providing patients with sickness leave is harmful/beneficial, good/bad, pleasant /unpleasant (for me), worthless/useful; I trust the intentions of patients who request for sickness leave during outpatient consultations (strongly disagree/strongly agree). The scores for both measurements ranged from 1 to 7 (1 = strongly disagree, 7 = strongly agree).

The indirect measure of attitude was designed from the themes derived from the open-ended questions during the pilot study. The arithmetic sum of the belief-based measure (seven-point likely/unlikely dimension) and corresponding outcome evaluations (seven-point extremely undesirable/desirable and not important/important dimension) determined the indirect measure of attitude. The scores for this measurement ranged from −84 to 84.

## Subjective norm

The direct measure of subjective norm involved inquiry using four items in the TPB construct: ‘Most people who are important to me think that (1 = should not to 7 = should) be strict in providing sickness leave to patients coming for outpatients clinic consultation; ‘It is expected of me to provide sickness leave to patients seeking outpatient clinic consultations’ ‘I feel under social pressure to provide sickness leave to patients who have come for outpatient; ‘People who are important to me recommend to be strict in providing sickness leave to patients who have come for outpatient consultations’. The scores for this measurement ranged from 1 to 7 (1 = strongly disagree to 7 = strongly agree).

A belief-based measure of subjective norm was derived from the summed response of 6 normative beliefs (4 injunctive and 2 descriptive) and motivations to comply. The items in the normative belief construct analyzed the factors that significant others would feel about the idea of providing sickness leave to patients. Motivation to comply was assessed based on willingness to provide sickness leave relative to the opinions of others. All items had a seven-point dimension (disapprove/approve, should not/should, avoid giving/do not avoid giving, not at all/very much). The scores for this measurement ranged from −84 to 84.

## Perceived behavioural control

The direct measure of perceived behavioural control was obtained by summing up responses to four items under the construct. These included a seven-point dimension assessment of self-efficacy: ‘The decision for me to provide my patients with sickness leave is easy or difficult (1 = difficult, 7 = easy)’, ‘I am confident that I could provide patients with sickness leave appropriately based on the consultation’ and controllability: ‘The decision to provide sickness leave is beyond my control’, ‘Whether I provide sickness leave or not is entirely up to me’. The scores for this measurement ranged from 1 to 7 (1 = strongly disagree to 7 = strongly agree).

A belief-based measure of perceived control was derived from the summed response of six beliefs influencing performance and ability to perform. The items for the influencing performance construct analyzed external factors that determined the respondent’s act of providing sickness leave to patients. The ability to perform construct assessed the willingness to provide sickness leave under the influence of the external factors. All items had a seven-point dimension (unpleasant/pleasant, disagree/agree). The scores for this measurement ranged from −84 to 84.

### Statistical analysis

#### Data management

All acquired data were entered into the Statistical Package for the Social Sciences program (SPSS version 20; IBM Corp., USA) and Statistics and Data Analysis program (STATA version 13.0; STATA Corp. USA). The items with negative endpoints were recoded to reflect actual scores [22, 23]. The data set was examined to account for missing or invalid values to ensure correct administration.

#### Reliability and validity

An exploratory factor analysis (EFA) was conducted to evaluate the loading variables using the principal component analysis (PCA) method. The oblique method of rotation (Direct Oblimin) was utilized for extraction of factors of the theoretical components via PCA. With the results of the exploratory factor analysis, the optimal number of factors to retain was obtained using parallel analysis. Subsequently, a confirmatory factor analysis (CFA) and structural equation modelling (SEM) was performed to assess the overall goodness of fit of the structural model.

Internal consistency and construct validity was determined in phases. Firstly, the internal consistency between items in the scales measuring behavioural intentions and the direct measures of attitude, subjective norm and perceived control was examined. A Cronbach’s alpha value above 0.7 was considered acceptable [24]. Once the reliability analysis had been completed, the themes were recoded into one composite variable measuring each construct (in total for variables) with a possible range of 1–7 [23].

Subsequently, the relationship between the theoretical constructs were explored using bivariate correlations (Pearson *R* test). It is hypothesized that the direct TPB measures would positively correlate with intention and are inter-related as they are not seen as categories independent of each other [22].

The relationship between indirect and direct measurements of the same constructs were then determined. By weighting belief statements based on the score of the relevant outcome evaluation, new variables were created into a new set of weighted beliefs by multiplying each behavioural statement with the corresponding behavioural outcome evaluation. In contrast to the direct measures, the indirect measures consist of both negative and positive beliefs because ‘people can quite logically hold negative and positive beliefs for the same behavior’ [23]. We performed a test-retest reliability to determine the temporal stability of the indirect measures [23].

Finally, we measured correlations between each individual indirect item and direct measurements of the same construct in order to determine if the beliefs had been identified and appropriately measured. Overall composite scores were then summed for each of the three variables. The results were reported as Pearson’s *R*, with a *p*-value <0.05 considered statistically significant.

#### Descriptive statistics

Descriptive analysis was undertaken to describe background characteristics (age, gender, and experience at work) and the distribution (mean and standard deviation) of the measures.

#### Predictability of the developed model

SEM was performed to examine the overall potential of the questionnaire to predict intentions and to identify the predictive strength of each direct and indirect measurement in the form of regression weights. For all outcomes, a *p*-value <0.05 was considered statistically significant. The explanatory power of the model was obtained from the *R*^2^ squared value of the model through an equation level goodness-of-fit test.

## Results

### Descriptive statistics

Out of 666 invited participants, 327 primary care physicians did not take part in the study. Therefore the drop-out rate was at 49.1%. Of 339 eligible physicians, a total of 271 (79.9%) completed the questionnaires. The mean age of the respondents was 31.8 years (SD ± 4.47; range 26–55 years).There was an over-representation of female participants (74.2%) but this is consistent with the proportion of the study population. The primary care physicians had an average of 5.97 years of service experience (including prior experience in hospital setting). About half (48.7%) recorded 3–5 years of professional experience and about one-third (29.2%) had 6–10 years of experience. The physicians had an average of 2.84 (SD ± 1.29) years of working in a primary care setting. One-third (30.3%) of physicians had at least 1–2 years of primary care working experience.

The mean scores for intention to provide patients with sickness leave was low with a value of 3.2 (SD ± 1.2). For the direct measures, the mean for perceived behavioural control recorded the highest score (4.9) in comparison with other constructs. As for indirect measures, the mean for attitudes recorded the highest score (26.4) followed by subjective norm (24.0) and perceived behavioural control (14.6).

### Reliability of the constructs in the direct measure questionnaire

The Cronbach α values for intentions to provide sickness leave to patients and direct measurements (Table 1) ranged from poor to very good across the sample. The Cronbach α values were 0.68 for intentions, 0.77 for attitude 1, 0.83 for attitude 2, 0.35 for subjective norms, 0.53 for perceived behavioural control. The Cronbach α value of the intention scale increased to 0.77 when item 1 was removed. Similarly, the internal consistency of the subjective norm scale increased to 0.62 when items 13 and 14 were removed from the construct.

**Table 1:** Reliability and items statistics for the constructs of direct measures

Constructs and items of direct measures	Mean (SD)	Cronbach Alpha	Item total correlation
Construct A: Generalized Intention		0.68	
Item 1	3.71(1.73)		0.38
Item 2	3.04(1.44)		0.65
Item 3	2.87(1.51)		0.50
Construct B1: Attitude ATT1 (attitude of physicians providing sickness leaves to patients)		0.77	
Item 4	4.25(1.21)		0.70
Item 5	4.08(1.71)		0.60
Item 6	3.55(1.36)		0.42
Item 7	4.34(1.33)		0.60
Construct B2: Attitude ATT2 (attitude of physicians trusting the intention of patients)		0.83	
Item 8	3.70(1.39)		0.73
Item 9	3.68(1.38)		0.67
Item 10	3.36(1.34)		0.59
Item 11	3.86(1.35)		0.65
Construct C: Subjective Norms		0.35	
Item 12	3.08(1.58)		0.33
Item 13	3.33(1.66)		0.19
Item 14	4.44(1.73)		0.05
Item 15	3.62(1.57)		0.20
Construct D: Perceived Control		0.53	
Item 16	5.67(1.30)		0.40
Item 17	4.22(1.56)		0.23
Item 18	4.74(1.82)		0.32
Item 19	5.04(1.71)		0.34

### Dimensionality of the developed model

In the primary EFA, 6 factors with Eigenvalues above 1 were found and explained 67% of the variance. Then, the numbers of factors were limited to 5 as determined via parallel analysis. This model explained 61% of the variance. The KMO measure of sampling adequacy was 0.706 and the Bartlett’s test of sphericity was statistically significant (*p* < 0.01). Consistent with the reliability analysis, items 13 and 14 had cross loadings and therefore requires removal from the scale (Table 2).

**Table 2:** Pattern matrix for exploratory factor analysis for direct measures

Constructs and items of direct measures	Factor 1	Factor 2	Factor 3	Factor 4	Factor 5	Extraction communalities
Construct A: Generalized Intention						
Item 1	0.597					0.415
Item 2	0.767					0.662
Item 3	0.723					0.571
ConstructB1: Attitude ATT1 (attitude of physicians providing sickness leaves to patients)						
Item 4		−0.820				0.761
Item 5		−0.834				0.660
Item 6		−0.541				0.423
Item 7		−0.768				0.652
Construct B2: Attitude ATT2 (attitude of physicians trusting the intention of patients)						
Item 8			0.843			0.729
Item 9			0.825			0.677
Item 10			0.768			0.592
Item 11			0.792			0.651
Construct C: Subjective Norms						
Item 12				0.762		0.625
Item 13				–		0.400
Item 14				–		0.290
Item 15				0.830		0.692
Construct D: Perceived Control						
Item 16					0.725	0.527
Item 17					0.556	0.433
Item 18					0.565	0.495
Item 19					0.688	0.512

### Fitness of the model

By performing CFA, the TPB model for direct measures fulfilled the indices required for a goodness of fit [root mean square error of approximation (RMSEA) < 0.01; comparative fit index (CFI) = 1.00; Tucker-Lewis index (TLI) = 1.00].

### Correlation between direct and indirect measures of the questionnaire

The correlation between indirect and direct constructs were statistically significant with a *p*-value of <0.01 (Table 3).

**Table 3:** Correlation coefficients (Pearson R) of intentions, direct and indirect measures

	Population (*n* = 271) Correlation coefficient (Pearson R)				
	Direct measures				
	Intentions	ATT1	ATT2	SN	PBC
Direct measures					
Direct attitude (ATT1)	0.32^a^	–			
Direct attitude (ATT2)	0.17^a^	–	–		
Direct subjective norm (SN)	0.20^a^	0.04	−0.10	–	
Direct perceived behavioural control (PBC)	−0.08	0.02	−0.02	−0.14^b^	–
Indirect measures					
Indirect attitude (ATT)	0.21^a^	0.30^a^	0.22^a^		
Indirect subjective norm (SN)	0.25^a^			0.15^b^	
Indirect perceived behavioural control (PBC)	−0.11				−0.21^a^

^a^Correlation is significant at the 0.01 level (2-tailed)

^b^Correlation is significant at the 0.05 level (2-tailed)

### Predictive strength of direct measures on intention to provide sickness leave

**Fig. 3:**
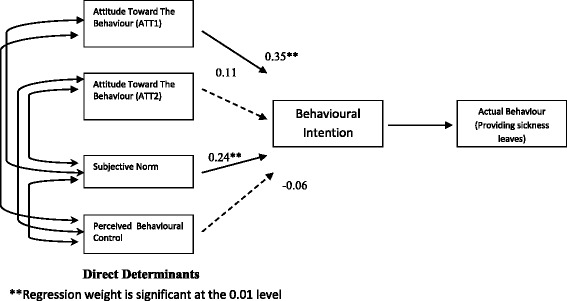
Path diagram of TPB model of intention to provide sickness certification to patients amongst primary care physicians

The path analysis conducted via SEM for direct measures (Fig. 3) showed that attitude ATT1 was the strongest predictor of intention to provide sickness leave to patients (β = 0.35, *p* < 0.01), followed by subjective norms (β = 0.24, *p* < 0.01). The regression weights for attitude ATT1 and subjective norm were statistically significant. However, this was not statistically significant for perceived behavioural control. An equation level of goodness-of-fit revealed that the three direct measurements accounted for 14.8% of the variation in the intention to provide sickness leave.

### Temporal stability of indirect measures

The correlation between composite scores for all three constructs (attitude, *r* = 0.334; subjective norm, *r* = 0.490; perceived norm, *r* = 0.370) of the indirect measures after a 2 week interval was adequate with a *p-*value of less than 0.05.

### Correlation with demographics

Only years of professional experience as a physician correlated with the intention to provide sickness leave (*p* < 0.05). Perceived behavioural control was found to be correlated to age (*p* < 0.01), professional years of experience (*p* < 0.01) and clinical years in primary care (*p* < 0.05).

## Discussion

### Main findings and comparison with literature

This study is the first attempt in this region to address the provision of sickness leave among primary care physicians using the TPB model. From a regional perspective, a thorough literature review reveals only two other studies pertaining to sickness leave in Malaysia [25, 26]. These studies lack the utilization of a robust psychological framework and focused primarily on descriptive evaluations of sickness absenteeism among employees [25, 26]. Similarly, most studies were also devoid of a suitable theoretical framework to enhance research accuracy [2–4, 11, 13, 27]. A core strength of this study lies in the basis of its theoretical approach to examine the intentions of primary care physicians in terms of providing sickness leave.

The total variance explained of the 5-factor solution of the TPB model for sickness certification behaviour amongst primary care physicians was good (>60%) [28]. Loading of all items in the model was optimal (>0.5) [29]. The model also demonstrated goodness of fit as evidenced by the RMSEA index (<0.06) and CFI (>0.95) [29]. The internal reliability of intentions and direct measures were adequate except for the perceived behavioural control construct. Consequently, the correlations between the constructs that were hypothesized to be theoretically-related were satisfactory except for perceived behavioural control. Therefore, the scale for perceived behavioural control should be omitted from the TPB theoretical model. On the other hand, the regression weight between intentions and the direct measures of subjective norm showed statistical significance despite a low Cronbach value of 0.62. This evidence suggests that although the subjective norm construct fits well with the TPB model of sickness leave provision by primary care physicians, items 13 and 14 were not relevant to the current set of participants in this study (as evidenced by poor total item correlation).

The results of this study indicate that the TPB variables, namely, attitude towards the behaviour and subjective norm are important factors associated with the primary care physician’s intention to provide sickness leave for patients. Perceived behavioural control poorly predicted behavioural intentions. One probable explanation for this is that most primary care physicians are confident in providing sickness leave to patients as this occurs on a daily basis in their practice. As the provision of sickness leave to patients becomes habitual, actual physician intent may play a lesser role.

### Strengths and limitations

In this study, bivariate correlations between direct and indirect measures were statistically significant but had low to average *r* values. Therefore it can be deduced that although the indirect measures was adequately constructed and covered the breadth of all measured constructs in the TPB model, these composites scores only had modest influence (based on effect size) on direct measures.

Although our study showed theoretical support for the use of the TPB model in the analysis of sickness leave in primary care, the study was limited by the low variance of overall explanation of the intentions to provide sickness leave (*r*^*2*^ = 14.8%). Therefore, we hypothesize that intentions could also be influenced by several factors not gauged in this study, 1) knowledge and skills to provide sickness leave, 2) salience of the act of providing sickness leave, 3) environmental constraints and 4) habitual aspect of providing sickness leave [21]. Thus, an integrated behavioural model [21] could be used to help elicit responses of latent variables that possibly remained unaddressed.

Another limitation of the study was that the data were cross-sectional. However, the reliability of the questionnaire was strengthened by conducting test-retest reliability which demonstrated adequate temporal stability. Future research endeavours should focus on a prospective study design using the TPB framework to allow the assessment of actual behaviour of providing sickness to patients not assessed in this study.

## Conclusion

Attitude was a strong predictor of intentions to provide sickness leave in this study, followed by subjective norm. Thus, attitudes and subjective norm should be considered as potential constructs for possible behavioural intervention. As TPB serves only to identify targets for intervention, other methods should be utilized to increase the likelihood of behaviour performance. This can be undertaken in the form of persuasive communication strategies that could help reduce conflict and aid decision making to provide sickness leave to patients among primary care physicians [21]. For example, one method of intervention would be the identification and training of popular opinions leaders (state/district health administrators, family medicine specialists or clinic administrator) to have conversations with peers (primary care physicians) and to model themselves as having adopted the behaviour changes being advocated from the results of the study (in this case beliefs based on the attitude and subjective norm construct) [21].

This study identifies predictors of intentions in providing sickness leave. However, intentions alone may be insufficient to determine behaviour due to an intention-behaviour gap [30]. Future studies should assess the efficacy of intention implementations strategies in increasing actual behaviour of performing an intention [30]. Finally, a larger study should be conducted in various other clinical settings to provide greater understanding of the variation that exists in physician intent to provide sickness leave to patients.

This findings could assist relevant agencies to facilitate the creation of policies that may help regulate the provision of sickness leave. This initiative can be achieved by adhering to a set of guidelines or organizational directives to alleviate the work burden of sickness leave tasks faced by physicians in Malaysia.

## Additional files

Additional file 1: Questionnaire 1 (Q1) Direct measurement of the act of providing sickness leaves to patients. (PDF 402 kb)

Additional file 2: Questionnaire 2 (Q2) Indirect measurement of the act of providing sickness leaves to patients. (PDF 496 kb)

## Abbreviations

ATT1: Direct measures of attitude of physicians providing sickness leaves to patients
ATT2: Direct measures of attitude of physicians trusting the intention of patients
CFI: Comparative fit index
EFA: Exploratory factor analysis
KMO: Kaiser-Meyer-Olkin test for sampling adequacy
PCA: Principal confirmatory analysis
Q1: Questionnaire 1 (Direct measures)
Q2: Questionnaire 2 (Indirect measures)
RMSEA: Root mean square error of approximation
SEM: Structural equation modelling
SPSS: Statistical Package for the Social Sciences program
STATA: Statistics and Data Analysis program
TLI: Tucker-Lewis index
TPB: Theory of Planned Behaviour

## References

[1] Wahlström R, Alexanderson K (2004). Swedish council on technology assessment in health care (SBU): chapter 11 - physicians’ sick-listing practices. Scand J Public Health Suppl.

[2] Winde LD, Alexanderson K, Carlsen B, Kjeldgård L, Wilteus AL, Gjesdal S (2012). General practitioners’ experiences with sickness certification: a comparison of survey data from Sweden and Norway. BMC Fam Pract.

[3] Lindholm C, Arrelöv B, Nilsson G, Löfgren A, Hinas E, Skånér Y (2010). Sickness-certification practice in different clinical settings; a survey of all physicians in a country. BMC Public Health.

[4] Foley M, Thorley K, Von Hout MC (2013). Sickness certification difficulties in Ireland -- a GP focus group study. Occup Med.

[5] Hussey S, Hoddinott P, Wilson P, Dowell J, Barbour R (2004). Sickness certification system in the United Kingdom: qualitative study of views of general practitioners in Scotland. BMJ.

[6] Swartling MS, Hagberg J, Alexanderson K, Wahlstrom RA (2007). Sick-listing as a psychosocial work problem: a survey of 3997 Swedish physicians. J Occup Rehabil.

[7] Roope R, Parker G, Turner S (2009). General practitioner’s use of sickness certificates. Occup Med.

[8] Meershoek A, Krumeich AVR (2007). Judging without criteria? Sickness certification in Dutch disability schemes. Soc Heal Illn.

[9] Bollag U, Rajeswaran A, Ruffieux C, Burnand B (2007). Sickness certification in primary care – the physician’s role. Swiss Med Wkly.

[10] Krohne K, Brage S (2007). New rules meet established sickness certification practice: a focus-group study on the introduction of functional assessments in Norwegian primary care. Scand J Prim Health Care.

[11] Engblom M, Nilsson G, Arrelöv B, Löfgren A, Skånér Y, Lindholm C (2011). Frequency and severity of problems that general practitioners experience regarding sickness certification. Scand J Prim Health Care.

[12] Löfgren A, Hagberg J, Arrelöv B, Ponzer S, Alexanderson K (2007). Frequency and nature of problems associated with sickness certification tasks: a cross-sectional questionnaire study of 5455 physicians. Scand J Prim Health Care.

[13] Von Knorring M, Sundberg L, Löfgren A, Alexanderson K (2008). Problems in sickness certification of patients: a qualitative study on views of 26 physicians in Sweden. Scand J Prim Health Care.

[14] Chew CA (1997). Benefits of back pain. Fam Pract.

[15] Wynne-Jones G, Mallen CD, Main CJ, Dunn KM (2010). What do GPs feel about sickness certification? A systematic search and narrative review. Scand J Prim Health Care.

[16] Shiels C, Gabbay M, Hillage J (2016). Recurrence of sickness absence episodes certified by general practitioners in the UK. Eur J Gen Pract.

[17] Hubertsson J, Englund M, Hallgårde U, Lidwall U, Löfvendahl S, Petersson IF (2014). Sick leave patterns in common musculoskeletal disorders–a study of doctor prescribed sick leave. BMC Musculoskelet Disord.

[18] Kankaanpää AT, Putus TM, Tuominen RJ (2014). Factors affecting sick leave prescribing in occupational health care: a survey based on hypothetical patient cases. BMC Health Serv Res.

[19] Tellnes G (1989). Sickness certification in general Practice : a review. Fam Pract.

[20] Grol RP, Bosch MC, Hulscher MEJL, Eccles MP, Wensing M (2007). Planning and studying improvement in patient care: the use of theoretical perspectives. Milbank Q..

[21] Glanz K, Rimer BKVK (2015). Health behaviour: theory, research and practice.

[22] Ajzen I (1991). The theory of planned behavior. Organ Behav Hum Decis Process.

[23] Francis J, Eccles MP, Johnston M, Walker A, Grimshaw J, Foy R (2004). Constructing questionnaires based on the theory of planned behaviour: a manual for health services researchers.

[24] Nunnaly JC (1978). Psychometric theory.

[25] Indran SK, Rampal KG, Ainsah O (1995). Absenteeism in the workplace, Klang Valley, Malaysia-preliminary report. Asia Pacific J Public Heal.

[26] Saroja KI, Ramphal KG, Kasmini K, Ainsah O, Bakar OC (1999). Trends in absenteeism rates following psychological intervention-preliminary result. Singap Med J.

[27] Grimshaw J, Campbell M, Eccles M, Steen N (2000). Experimental and quasi-experimental designs for evaluating guideline implementation strategies. Fam Pract.

[28] Hair JF, Black WC, Babin BJ, Anderson RE (2013). Multivariate Data Analysis.

[29] Tabachnick BG, Fidell SL (2007). Using multivariate statistics.

[30] Stroebe W (2011). Social psychology and health.

