# Supplementary material for: Assessing predictors of intention to prescribe sick leave among primary care physicians using the theory of planned behaviour
Source: BMC Fam Pract. 2018 Jan 16;19:18. doi: 10.1186/s12875-017-0690-5 (PMC5771020; doi:10.1186/s12875-017-0690-5)
Supplement: Supplementary file 1 — Questionnaire 1 (Q1) Direct measurement of the act of providing sickness leaves to patients. (PDF 402 kb) [file 12875_2017_690_MOESM1_ESM.pdf]

Clinician Survey In Providing Sickness Leaves To Patients

(Questionnaire 1)

This survey is part of a larger project aimed at better understanding the beliefs and attitudes that contribute to the behaviour in providing medical certification for patients. By gathering information from many medical officers, we hope to learn what factors are most important in affecting the intention in providing medical certification to patients.

This booklet contains a series of brief questionnaires that take about 15 minutes to complete. Please answer EVERY question in the booklet. Instructions on how to respond to the different questionnaires in the booklet are provided at the top of each page. Please note that there are no right or wrong answers, just what YOU think and how YOU perceive your work situation.

All the information that you provide in this session will be held in confidentiality. Your responses will be kept by the researchers, and we will aggregate responses from all interviews so that no one individual will be identifiable.

Subject Number __ __ __
Clinic ________

Background Information

For each question below, either write in your answer in the space given.

1. What is your profession? (medical officer/family medicine specialist)

2. How many years of experience do you have working in this profession? _____ yrs

3. Are you ____ Male or ____ Female?

4. How long have you been working in this clinic? ____ years and ____ months

5. What is your age? _________ years old

Generalised Intention

Directions: use the scale below to indicate how much you agree or disagree with each statement by circling the number that best corresponds to your answer in the space next to the question number. Remember there are no right or wrong answers, only what is TRUE of you.

Question Format	Response Format
I expect to provide patients MCs during outpatient consultation	*Strongly Disagree* **1 2 3 4 5 6 7** *Strongly Agree*
I want to provide patients with MCs during an outpatient consultation	*Strongly Disagree* **1 2 3 4 5 6 7** *Strongly Agree*
I intend to provide patients with MCs during an outpatient consultation	*Strongly Disagree* **1 2 3 4 5 6 7** *Strongly Agree*

Attitude

Directions: use the scale below to indicate how much you agree or disagree with each statement by circling the number that best corresponds to your answer in the space next to the question number. For the indirect measurement section, answer briefly on what you think is your interpretation of your working environment. Remember there are no right or wrong answers, only what is TRUE of you.

A patient attends the outpatient clinic for routine consultation. The target behaviour is providing sickness certification to the patient:

Question Format	Response Format
Providing patients with MCs is	*harmful* ***1 2 3 4 5 6 7*** *beneficial*<br>*good* ***1 2 3 4 5 6 7*** *bad*<br>*pleasant (for me)* ***1 2 3 4 5 6 7*** *unpleasant (for me)*<br>*not important* ***1 2 3 4 5 6 7*** *important*
Trusting the intentions of patients who request for MCs during outpatient consultations is	harmful **1 2 3 4 5 6 7** beneficial<br>good **1 2 3 4 5 6 7** bad<br>pleasant (for me) **1 2 3 4 5 6 7** unpleasant (for me)<br>worthless **1 2 3 4 5 6 7** useful

Subjective Norm

Directions: use the scale below to indicate how much you agree or disagree with each statement by circling the number that best corresponds to your answer in the space next to the question number. For the indirect measurement section, answer briefly on what you think is your interpretation of your working environment. Remember there are no right or wrong answers, only what is TRUE of you.

A patient presents with to the outpatient clinic for consultation. The target behaviour is providing the patient with MCs:

Question Format	Response Format
Most people who are important to me think that **..............** be strict in providing MCs to patients come for outpatient clinic consultation	*I should not **1 2 3 4 5 6 7** I should*
It is expected of me to provide MCs to patients who come for outpatient clinic consultations	*Strongly Disagree* **1 2 3 4 5 6 7** *Strongly Agree*
I feel under social pressure to provide MCs to patients who have come for outpatient consultations.	*Strongly Disagree* **1 2 3 4 5 6 7** *Strongly Agree*
People who are important to me recommend that I be strict in providing MCs to patients who have come for outpatient consultations.	*Strongly disagree **1 2 3 4 5 6 7** Strongly agree*

Perceived Control

Directions: use the scale below to indicate how much you agree or disagree with each statement by circling the number that best corresponds to your answer in the space next to the question number. For the indirect measurement section, answer briefly on what you think is your interpretation of your working environment. Remember there are no right or wrong answers, only what is TRUE of you.

Question Format	Response Format
I am confident that I could provide patients with MCs appropriately based on the consultation	*Strongly Disagree* **1 2 3 4 5 6 7** *Strongly Agree*
The decision for me to provide my patients with MCs is	*Easy* ***1 2 3 4 5 6 7*** *Difficult*
The decision to provide MCs is beyond my control	*Strongly Disagree* **1 2 3 4 5 6 7** *Strongly Agree*
It is entirely up to me whether I provide MC or not	*Strongly disagree* ***1 2 3 4 5 6 7*** *Strongly Agree*

-Infinite Thanks for Your Time And Effort-
