# Supplementary material for: Assessing predictors of intention to prescribe sick leave among primary care physicians using the theory of planned behaviour
Source: BMC Fam Pract. 2018 Jan 16;19:18. doi: 10.1186/s12875-017-0690-5 (PMC5771020; doi:10.1186/s12875-017-0690-5)
Supplement: Supplementary file 2 — Questionnaire 2 (Q2) Indirect measurement of the act of providing sickness leaves to patients. (PDF 496 kb) [file 12875_2017_690_MOESM2_ESM.pdf]

Clinician Survey In Providing Sickness Leaves To Patients

(Questionnaire 2)

This survey is part of a larger project aimed at better understanding the beliefs and attitudes that contribute to the behaviour in providing medical certification for patients. By gathering information from many medical officers, we hope to learn what factors are most important in affecting the intention in providing medical certification to patients.

This booklet contains a series of brief questionnaires that take about 15 minutes to complete. Please answer EVERY question in the booklet. Instructions on how to respond to the different questionnaires in the booklet are provided at the top of each page. Please note that there are no right or wrong answers, just what YOU think and how YOU perceive your work situation.

All the information that you provide in this session will be held in confidentiality. Your responses will be kept by the researchers, and we will aggregate responses from all interviews so that no one individual will be identifiable.

Subject Number __ __ __
Clinic ________

Generalised Intention

Directions: use the scale below to indicate how much you agree or disagree with each statement by circling the number that best corresponds to your answer in the space next to the question number. Remember there are no right or wrong answers, only what is TRUE of you.

Question Format	Response Format
I plan to provide patients MCs during outpatient consultation	*Disagree **1 2 3 4 5 6 7** Agree*
I want to provide patients with MCs during an outpatient consultation	*Disagree **1 2 3 4 5 6 7** Agree*
I intend to provide patients with MCs during an outpatient consultation	*Disagree **1 2 3 4 5 6 7** Agree*

Attitude

Directions: use the scale below to indicate how much you agree or disagree with each statement by circling the number that best corresponds to your answer in the space next to the question number. Remember there are no right or wrong answers, only what is TRUE of you.

Question Format	Response Format
I provide MCs so that it will allow patients to recover from their illness	*Unlikely **1 2 3 4 5 6 7** Likely*
I hesitate to provide MCs to patients as it promotes MC seeking behaviour	*Unlikely **1 2 3 4 5 6 7** Likely*
The decision to provide MCs is influenced by the patient's behaviour and attitude towards me	*Unlikely **1 2 3 4 5 6 7** Likely*
I provide MCs based on the condition of the patient after a clinical assessment	*Unlikely **1 2 3 4 5 6 7** Likely*
I believe that providing MCs to patients reduces their work productivity	*Unlikely **1 2 3 4 5 6 7** Likely*
I believe that patients have genuine intentions when they request for an MC	*Unlikely **1 2 3 4 5 6 7** Likely*

Question Format	Response Format
Allowing patients to recover from their illness is:	*Extremely undesirable* ***-3 -2 -1 0 +1 +2 +3*** *Desirable*
MC seeking behaviour to me is:	*Extremely undesirable* ***-3 -2 -1 0 +1 +2 +3*** *Desirable*
Proper patient behaviour or attitude is to me:	*Extremely undesirable* ***-3 -2 -1 0 +1 +2 +3*** *Desirable*
The general condition of a patient to me is:	*Not important* ***-3 -2 -1 0 +1 +2 +3*** *Important*
Reduction in patient's work productivity is:	*Extremely undesirable* ***-3 -2 -1 0 +1 +2 +3*** *Desirable*
The genuine intention of patients is to me	*Extremely undesirable* ***-3 -2 -1 0 +1 +2 +3*** *Desirable*

Subjective Norms

Directions: use the scale below to indicate how much you agree or disagree with each statement by circling the number that best corresponds to your answer in the space next to the question number. Remember there are no right or wrong answers, only what is TRUE of you.

Question Format	Response Format
Clinical specialists would **.............** of me providing MCs to patients during outpatient consultations	*Disapprove* ***-3 -2 -1 0 +1 +2 +3*** *Approve*
Patients think I **................** provide them MCs during outpatient consultation.	*Should not* ***-3 -2 -1 0 +1 +2 +3*** *Should*
Health managers (i.e. Medical Officer In Charge Of Clinic/State Director/District director) would **................** of me providing MCs to patients during outpatient consultations.	*Disapprove* ***-3 -2 -1 0 +1 +2 +3*** *Approve*
Employers of the patient think I **..............** provide them MCs during outpatient consultation	*Should not* ***-3 -2 -1 0 +1 +2 +3*** *Should*
Other colleagues usually **...............** MCs during outpatient consultation	*Avoid giving* ***-3 -2 -1 0 +1 +2 +3*** *Give*
Our organization (the clinic/state/district health department) **................** us to provide MCs to patients during outpatient consultation	*Does not encourage* ***-3 -2 -1 0 +1 +2 +3*** *Encourage*

Question Format	Response Format
What the clinical specialist thinks I should do matters to me	*Not at all* ***1 2 3 4 5 6 7*** *Very much*
Patient’s approval of my practice is important to me	*Not at all* ***1 2 3 4 5 6 7*** *Very much*
What the health managers think I should do matters to me	*Not at all* ***1 2 3 4 5 6 7*** *Very much*
I take seriously the employers need to maintain work productivity of the patient	*Disagree* ***1 2 3 4 5 6 7*** *Agree*
Doing what other colleagues do is important to me	*Not at all* ***1 2 3 4 5 6 7*** *Very Much*
The need to follow organizational norms/culture is important to me	*Not at all* ***1 2 3 4 5 6 7*** *Very Much*

Perceived Control

Directions: use the scale below to indicate how much you agree or disagree with each statement by circling the number that best corresponds to your answer in the space next to the question number. Remember there are no right or wrong answers, only what is TRUE of you.

Question Format	Response Format
Patient's behaviour during a consultation when requesting MC can be (for me)	*Unpleasant* ***1 2 3 4 5 6 7*** *Pleasant*
Organizational pressure (i.e. Medical Officer In Charge Of Clinic/State Director/District director) influences my decision making of providing MCs to patients	*Disagree* ***1 2 3 4 5 6 7*** *Agree*
MCs are for patients with appropriate clinical presentation	*Disagree* ***1 2 3 4 5 6 7*** *Agree*

Question Format	Response Format
I find providing MCs to patients confrontational	*Disagree* **-3 -2 -1 0 +1 +2 +3** *Agree*
I have very little or no autonomy over providing MCs to patients	*Disagree* **-3 -2 -1 0 +1 +2 +3** *Agree*
I find it difficult to provide MCs to patients with unclear clinical symptoms or signs	*Disagree* **-3 -2 -1 0 +1 +2 +3** *Agree*

-Infinite Thanks For Your Time And Effort-
